# Neoadjuvant Chemotherapy Combined with Breast-Conserving Surgery in the Treatment of Triple-Negative Breast Cancer

**DOI:** 10.1155/2022/7847889

**Published:** 2022-05-26

**Authors:** Xiaoyu Zhang, Huixin Li, Feng Wu, Dan Sun, Hengle Zhang, Lijun Jin, Xiaoning Kang, Zunyi Wang

**Affiliations:** ^1^Cangzhou Central Hospital, Cangzhou, China; ^2^Hebei Medical University, Cangzhou Central Hospital Affiliated to Hebei Medical University, Cangzhou, China

## Abstract

**Objective:**

To study the clinical efficacy and quality of life of neoadjuvant chemotherapy combined with breast-conserving surgery in the treatment of triple-negative breast cancer.

**Methods:**

A retrospective analysis of 100 patients with triple-negative breast cancer was performed from May 2012 to April 2017. The patients were divided into an observation group and a control group according to different treatment methods, with 50 cases in each group. The control group received AC-T sequential chemotherapy after breast-conserving surgery, and the observation group received AC-T sequential chemotherapy before breast-conserving surgery (neoadjuvant). The operation time, postoperative immune function, postoperative tumor markers, postoperative efficacy, and postoperative complications of the two groups of patients were statistically analyzed, and the quality of life of the two groups of patients 1 year after the operation was compared.

**Results:**

Compared with the control group, the operation time and blood loss of the observation group were significantly reduced, and the difference was statistically significant (*P* < 0.05). The observation group produced significantly higher total effective rate after treatment (82.00% vs. 56.00%) (*P* < 0.05). The observation group exhibited superior immune function indexes CD3, CD4, and CD8 after operation when compared with the control group (*P* < 0.05). There was no significant difference in serum tumor marker levels between the two groups before surgery and after surgery (both *P* > 0.05). Three days after operation, the levels of procalcitonin (PCT) and TNF-*α* in the observation group were lower than those in the control group (*P* < 0.05). There was no significant difference in the local recurrence rate, distant metastasis rate, and 3-year survival rate between the two groups (*P* > 0.05); however, the postoperative complication rate of the observation group was 6.00%, which was significantly lower than that of the control group (30%) (*P* < 0.05). The overall health, physiological function, physiological function, and body pain of the observation group were significantly higher than those of the control group (*P* < 0.05).

**Conclusion:**

Neoadjuvant chemotherapy combined with breast-conserving surgery for triple-negative breast cancer can not only improve the therapeutic effect of patients and reduce the incidence of postoperative adverse reactions but also significantly improve the quality of life of patients after surgery.

## 1. Introduction

With the acceleration of social pace and the increase of life pressure, the incidence of breast cancer intends to rise in young population [1]. And patients have an increasingly higher demand for treatment efficacy due to the enhancement of people's health awareness and the diagnosis [2]. Clinically, breast cancer is primarily treated with surgery, of which breast-conserving surgery is the most commonly used. On the basis of traditional radical surgery or modified radical surgery, breast-conserving surgery preserves the breasts of patients and satisfies the psychological needs of patients for aesthetics. Triple-negative breast cancer (TNBC) is a cancer where estrogen receptor (ER), progesterone receptor (PR), and human epidermal growth factor receptor 2 (HER-2) are all negative [3]. TNBC is characterized by high degree of malignancy and strong invasiveness, and endocrine therapy and targeted therapy are associated with easy metastasis, easy recurrence, and poor prognosis. Therefore, TNBC is a topical and challenging issue in clinical treatment [4]. Breast cancer is a systemic disease, and its tumor cells can metastasize to various organs in the body via blood circulation, and surgical treatment alone as a result cannot completely eliminate tumor cells [5]. Neoadjuvant therapy can also be used to reduce the extent of local therapy or reduce delays in initiating therapy [6]. Appropriate candidates for neoadjuvant therapy include patients with inflammatory breast cancer and those in whom residual disease may prompt a change in therapy [6]. In light of these, the present study investigated the clinical efficacy of neoadjuvant chemotherapy combined with breast-conserving surgery in TNBC patients and its effect on tumor markers and immune function, which is aimed at improving the clinician's diagnosis and treatment of breast cancer.

## 2. Materials and Methods

### 2.1. General Information

A retrospective analysis of 100 TNBC patients confirmed by pathology and immunohistochemistry in our hospital from May 2012 to April 2017 was conducted. All patients were diagnosed with breast cancer by needle biopsy and pathological biopsy, and they did not receive any chemotherapy, hormone therapy, and radiotherapy, and routine examination showed no distant metastasis and organ damage. The study protocol was reviewed and authorized by the hospital ethics committee, No.2304/9991.

Inclusion criteria: (1) All met the diagnostic criteria for breast cancer in the *Guidelines and Specifications for Breast Cancer Diagnosis and Treatment of the Chinese Anti-Cancer Association* (2008 Edition) [7], and the pathological diagnosis was IIb and III; (2) biopsy by mammotome or hollow needle was performed before implementation; and (3) complete pathological results and lymph node metastasis results. This study was approved by the hospital ethics committee, and all patients signed informed consent.

Exclusion criteria are as follows: (1) positive expression of Her-2 and/or HR; (2) patients with severe heart, liver, kidney, and other organ dysfunction, acute and chronic infectious diseases, and other malignant tumors; (3) pregnant or lactating women; (4) those with allergic constitution and contraindications to the study drug; (5) those with disease progression during neoadjuvant chemotherapy; (6) those with an expected survival period of less than 3 months; and (7) those who were unwilling to sign the informed consent form. According to different treatment methods, 100 patients were allocated (1 : 1) into the control group and the observation group. The average age of patients in the control group was 50.06 ± 5.22 years old, and in the observation group was 49.84 ± 6.26 years old. The baseline data were homogeneous in the two groups, suggesting the feasibility of the study.

### 2.2. Intervention

The control group received AC-T sequential chemotherapy after breast-conserving surgery, and the observation group received AC-T sequential chemotherapy before breast-conserving surgery (neoadjuvant).

*Breast-conserving surgery*. The patient was placed in the supine position, routinely sterilized and draped, and treated with local wide excision of the tumor combined with axillary lymph node dissection or axillary sentinel lymph node biopsy, and the shape of the incision was designed according to the location of the tumor, and the tissue was frozen for pathological examination after surgery.

*AC-T sequential chemotherapy regimen*. AC: On day 1, doxorubicin hydrochloride liposome 30 mg/m^2^ was administered via intravenous injection and cyclophosphamide 600 mg/m^2^; 21 days was a cycle for 4 consecutive cycles of treatment. Sequential T: On day 1, docetaxel 80 mg/m^2^ was intravenously injected; 21 days was a cycle for 4 consecutive cycles of treatment.

### 2.3. Observation Indicators and Follow-Up Period

*Efficacy evaluation*. The postoperative therapeutic effects of the two groups of subjects were recorded and analyzed, including the following: operation time, intraoperative blood loss, surgical complication rate, 3-year local recurrence rate, and 3-year distant metastasis rate.

*Quality of life*. It was assessed according to the KPS score after treatment. The KPS score ranged from 0 to 100, with 0 being death and 100 being normal. The higher the score, the higher the quality of life. After treatment, the patients were evaluated according to the Functional Assessment of Cancer Treatment-Breast Cancer (FACT-B) score, and the score was 0-4 points. The higher the total score, the better the quality of life.

*Detection of tumor markers and immune indexes*. The fasting peripheral venous blood of all patients in the morning was drawn before and after treatment, respectively. Serum tumor markers were detected by electrochemiluminescence method; peripheral blood T lymphocyte subsets were determined by Beckman Coulter Epics XL flow cytometer and supporting kits. All operations were carried out in strict accordance with the instructions.

### 2.4. Evaluation Criteria

Markedly effective: the patient's visible lesions completely disappeared after examination; effective: the sum of the product of the two largest vertical diameters of the patient's lesions was reduced by more than 50%, and the clinical efficacy was improved; ineffective: the patient's condition after surgery has not been effectively controlled, and the lesions have metastasized, and local recurrence, lymphoid tissue, and distant metastasis have occurred. The total effective rate is the sum of the markedly effective and effective rates.

### 2.5. Statistical Analysis

All data analysis was performed with the SPSS 25.0 statistical software. Count data was expressed as percentage (%) and processed by chi-square test, and measurement data was expressed as mean ± standard deviation and verified via *t*-test. *P* < 0.05 indicated a statistically significant difference.

## 3. Results

### 3.1. Baseline Data

The two groups presented comparable baseline features such as age, course of disease, tumor diameter, and disease condition (*P* > 0.05) (see Table 1).

### 3.2. Intraoperative and Postoperative Indicators

Compared with the control group, the operation time, the blood loss, and the hospital stay of the observation group were significantly reduced, and the difference was statistically significant (*P* < 0.05) (see Table 2).

### 3.3. Treatment Effectiveness

The observation group produced significantly higher total effective rate after treatment (82.00% vs. 56.00%) (*P* < 0.05), as shown in Table 3.

### 3.4. Immune Function

The observation group exhibited superior immune function indexes CD3, CD4, and CD8 after operation when compared with the control group (*P* < 0.05), as shown in Table 4.

### 3.5. Serum Tumor Marker Levels

There was no significant difference in serum tumor marker levels between the two groups before surgery and after surgery (both *P* > 0.05) (see Table 5).

### 3.6. PCT and TNF-*α* Levels

Before surgery, the PCT and TNF-*α* were similar in the two groups (*P* > 0.05). Three days after operation, the levels of PCT and TNF-*α* in the observation group were lower than those in the control group (*P* < 0.05) (see Table 6).

### 3.7. Postoperative Adverse Reactions and Long-Term Prognosis

There was no significant difference in the local recurrence rate, distant metastasis rate, and 5-year survival rate between the two groups (*P* > 0.05); however, the postoperative complication rate of the observation group was 6.00%, which was significantly lower than that of the control group (30%) (*P* < 0.05) (see Table 7).

### 3.8. Quality of Life

The overall health, physiological function, physiological function, and body pain of the observation group were significantly higher than those of the control group (*P* < 0.05) (see Table 8).

## 4. Discussion

According to the latest data, there are annually about 270,000 new cases of breast cancer in China, and about 70,000 women die from breast cancer, and the figure continues to rise. How to prevent and treat breast cancer has become an urgent clinical problem [8–10]. Advanced breast cancer would be complicated by tumor cachexia syndrome and metastasize to distant sites, such as lung, bone, and liver, which seriously threatens the life safety of patients. Triple-negative breast cancer (TNBC) is defined as estrogen receptor (ER), progesterone receptor (PR), and human epidermal growth factor receptor 2 (HER-2). All are negative breast cancers [11–13]. TNBC is characterized by high degree of malignancy and strong invasiveness, poor endocrine therapy and targeted therapy, easy metastasis, easy recurrence, and poor prognosis [14]. Therefore, TNBC is a difficult point in clinical treatment and a hot spot of current research.

Breast-conserving surgery for breast cancer is performed by excising the mass and performing pathological biopsy at the incision site after surgery, which can determine the effect of cancer cell removal and maximize the control of postoperative recurrence [15]. However, studies have found [16] that after surgical treatment, severe edema of the upper extremity occurs, which affects the postoperative recovery effect; the incision is large, which is prone to infection, and the prognosis is poor. In recent years, studies have found [17–19] that preoperative neoadjuvant chemotherapy can induce apoptosis and differentiation of cancer cells, which is of positive significance for accelerating postoperative recovery and reducing complications. At the same time, it can effectively control recurrence, prolong the survival period of patients, and improve the quality of life [2]. Clinical practice shows that neoadjuvant chemotherapy plays an important role in improving the treatment effect before breast-conserving surgery for breast cancer [3, 20]. The results of this study show that preoperative neoadjuvant chemotherapy combined with breast-conserving surgery can effectively shorten the operation time and hospital stay and reduce the amount of intraoperative blood loss in patients compared with postoperative neoadjuvant chemotherapy. The results of this study also found that the total effective rate of patients receiving preoperative neoadjuvant chemotherapy combined with breast-conserving surgery was 82.00%, and the control group was 56.00%. The therapeutic effect of adjuvant chemotherapy combined with breast-conserving surgery is significantly higher than that of patients with postoperative neoadjuvant chemotherapy. The reason is that neoadjuvant chemotherapy combined with breast-conserving surgery, on the premise of breast-conserving the treatment process, first uses chemotherapy to control the spread and metastasis of cancer cells, then accurately determines the area where the cancer cells are located, and cuts them during the operation. To a certain extent, the clinical efficacy has been improved. Secondly, neoadjuvant chemotherapy has a certain killing effect on tumor cells, which reduces the activity of tumor tissue and reduces the spread of tumor cells during and after surgery. This study also shows that preoperative neoadjuvant chemotherapy can help improve the patient's immune function indicators, but the specific mechanism remains to be further studied.

In addition, the results of this study showed that there was no significant difference in the local recurrence rate, distant metastasis rate, and 5-year survival rate between the two groups; however, the postoperative complication rate of the observation group was 6.00% was significantly less than 30.00% of the control group. It can be seen that the postoperative safety of preoperative neoadjuvant chemotherapy in triple-negative breast cancer is higher than that of postoperative neoadjuvant chemotherapy, and it can also play a role in improving postoperative survival. At the same time, the 1-year follow-up results of all patients showed that the quality of life of patients receiving preoperative neoadjuvant chemotherapy combined with breast-conserving surgery, such as physiological function, physiological function, and physical pain, was higher than postoperative neoadjuvant chemotherapy. We speculated that neoadjuvant chemotherapy combined with breast-conserving surgery can control the patient's condition more effectively and relieve the patient's pain during the treatment process, offer great psychological comfort, and reduce psychological trauma.

In conclusion, preoperative neoadjuvant chemotherapy combined with breast-conserving surgery has better clinical efficacy. It reduces the incidence of postoperative adverse reactions, improves the safety of clinical treatment, and boosts the quality of life of postoperative patients.

## Figures and Tables

**Table 1:** Comparison of general data of the two groups of patients.

Group	*n*	Age (year)	Course of disease (d)	Tumor diameter (cm)	Tumor site (case)	
					Left	Right
Observation group	50	49.84 ± 6.26	33.42 ± 5.73	3.74 ± 0.98	35	15
Study group	50	50.06 ± 5.22	33.62 ± 5.95	3.69 ± 0.86	33	17
*χ* ^2^/*t*		0.191	0.171	0.272	0.184	
*P*		0.849	0.864	0.786	0.668	

**Table 2:** Comparison of intraoperative and postoperative indicators between the two groups of patients.

Group	*n*	Operation time (min)	Blood loss (ml)	Hospital stay (d)
Observation group	50	45.36 ± 7.75	103.63 ± 5.42	4.52 ± 1.20
Study group	50	72.75 ± 8.63	229.73 ± 5.39	8.66 ± 1.08
*χ* ^2^/*t*		16.688	116.641	18.134
*P*		≤0.001	≤0.001	≤0.001

**Table 3:** Comparison of effective rates between the two groups of patients.

Group	n	Markedly effective	Effective	Ineffective	Total effective rate
Observation group	50	21	20	9	41 (82.00)
Study group	50	15	13	22	28 (56.00)
*χ* ^2^					7.901
*P*					0.005

**Table 4:** Comparison of immune function between two groups of patients.

Group	*n*	CD3+ (%)		CD4+ (%)		CD8+ (%)	
		Before treatment	After treatment	Before treatment	After treatment	Before treatment	After treatment
Observation group	50	53.44 ± 3.65	71.42 ± 3.84	34.67 ± 2.86	42.84 ± 3.25	32.89 ± 3.59	39.93 ± 4.65
Study group	50	53.72 ± 3.32	52.07 ± 3.75	34.79 ± 2.91	32.87 ± 3.54	32.83 ± 3.25	34.15 ± 3.28
*t*		0.413	25.514	0.222	14.669	0.088	7.189
*P*		0.681	≤0.001	0.825	≤0.001	0.930	≤0.001

**Table 5:** Comparison of serum tumor marker levels before and after surgery in two groups of patients.

Group	*n*	TSGF (U/ml)		CEA (ng/ml)	
		Before treatment	After treatment	Before treatment	After treatment
Observation group	50	155.37 ± 11.03	63.54 ± 5.31	11.46 ± 1.43	3.46 ± 0.93
Study group	50	155.29 ± 11.52	63.49 ± 5.46	11.51 ± 1.47	3.48 ± 0.97
*t*		0.032	0.039	0.200	0.148
*P*		0.975	0.969	0.842	0.883

**Table 6:** Changes of PCT and TNF-*α* levels before and after surgery in the two groups of patients.

Group	*n*	PCT (ng/ml)		TNF-*α* (ng/ml)	
		Before treatment	After treatment	Before treatment	After treatment
Observation group	50	2.31 ± 0.18	4.61 ± 1.08	181.46 ± 21.43	173.54 ± 20.97
Study group	50	2.27 ± 0.25	6.27 ± 1.07	181.51 ± 21.47	193.46 ± 20.93
*t*		0.723	7.690	0.012	4.756
*P*		0.472	≤0.001	0.991	≤0.001

**Table 7:** Comparison of postoperative adverse reactions and long-term prognosis in the two groups of patients.

Group	*n*	Postoperative complication	Local recurrence	Distant metastasis	5-year survival
Observation group	50	3	5	7	43
Study group	50	15	7	6	41
*χ* ^2^		9.756	0.379	0.088	0.298
*P*		0.002	0.538	0.766	0.585

**Table 8:** Comparison of the quality of life of the two groups of patients at 1 year after surgery.

Group	*n*	Overall health	Physiological function	Physical function	Body pain
Observation group	50	83.31 ± 7.22	84.52 ± 8.13	82.87 ± 9.03	89.47 ± 8.09
Study group	50	74.86 ± 7.88	75.62 ± 8.83	70.81 ± 8.72	71.32 ± 8.86
*t*		5.590	5.249	6.789	10.693
*P*		≤0.001	≤0.001	≤0.001	≤0.001

## Data Availability

The datasets used during the present study are available from the corresponding author upon reasonable request.
